# Supplementary material for: Phytochemical Screening and Biological Activities of Lippia multiflora Moldenke
Source: Molecules. 2025 Jul 7;30(13):2882. doi: 10.3390/molecules30132882 (PMC12250754; doi:10.3390/molecules30132882)
Supplement: Supplementary file 1 [file molecules-30-02882-s001.zip › molecules-3696371-supplementary.pdf]

Article

Phytochemical Screening and Biological Activities of *Lippia multiflora* Moldenke

Dorcas Tlhapi [1,*], Ntsoaki Malebo [2], Idah Tichaidza Manduna [1], Monizi Mawunu [3,4] and Ramakwala Christinah Chokwe [5]

1 Centre for Applied Food Sustainability and Biotechnology, Faculty of Health and Environmental Sciences, Central University of Technology, Bloemfontein 9300, South Africa; imanduna@cut.ac.za
2 Centre for Innovation in Learning and Teaching, Central University of Technology, Bloemfontein 9300, South Africa; nmalebo@cut.ac.za
3 Department of Agronomy, Polytechnic Institute, Kimpa Vita University, Uíge P.O. Box 77, Angola; m.mawunu2000@gmail.com
4 Department of Biology, Faculty of Science and Technology, University of Kinshasa, Kinshasa P.O. Box 190, Congo
5 Department of Chemistry, College of Science Engineering and Technology, University of South Africa, Johannesburg 1710, South Africa; chokwrc@unisa.ac.za
* Correspondence: btlhapi@cut.ac.za

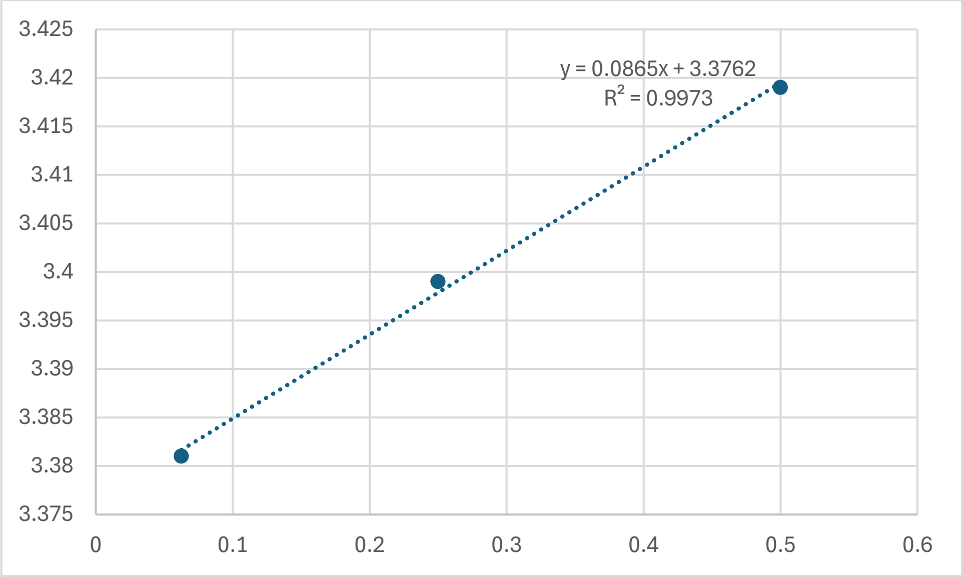

Figure S1. Regression equation of quercetin calibration curve.

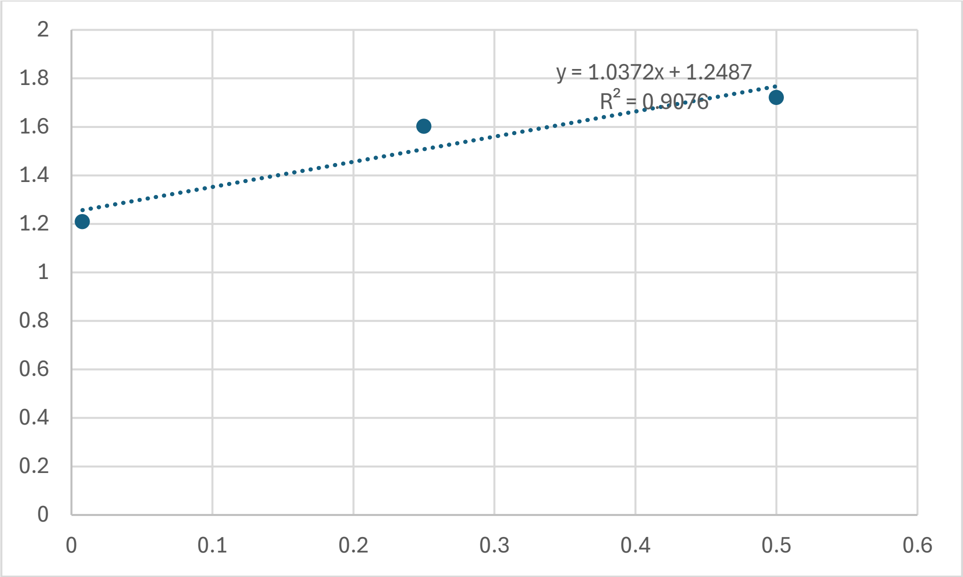

Figure S2. Regression equation of gallic acid calibration curve.

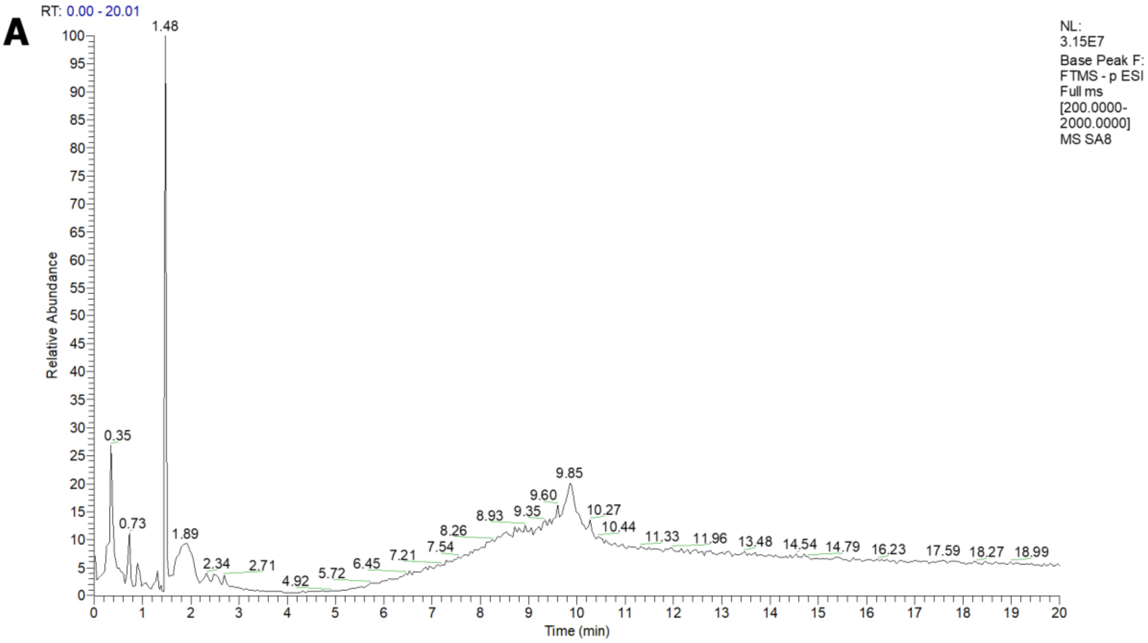

B

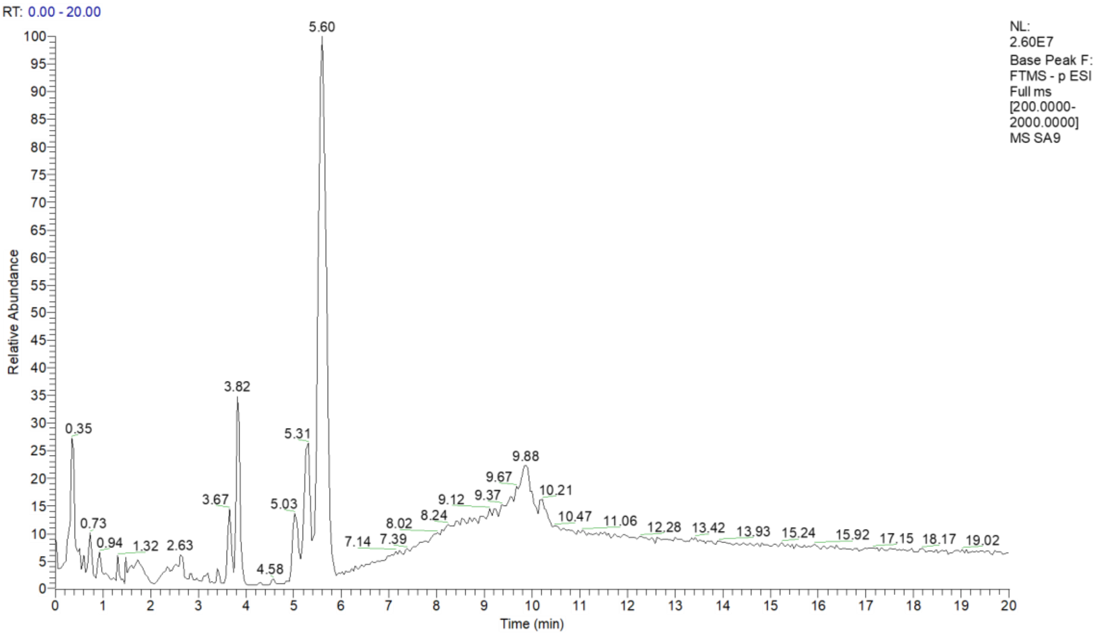
RT: 0.00 - 20.00
NL: 2.60E7
Base Peak F: FTMS - p ESI Full ms [200.0000-2000.0000] MS SA9
Relative Abundance
Time (min)
5.60
3.82
0.35
5.31
9.88
9.67
10.21
3.67
5.03
9.12
9.37
0.73
8.24
8.02
10.47
11.06
12.28
13.42
13.93
15.24
15.92
17.15
18.17
19.02
0.94
1.32
2.63
7.14
7.39
4.58

C

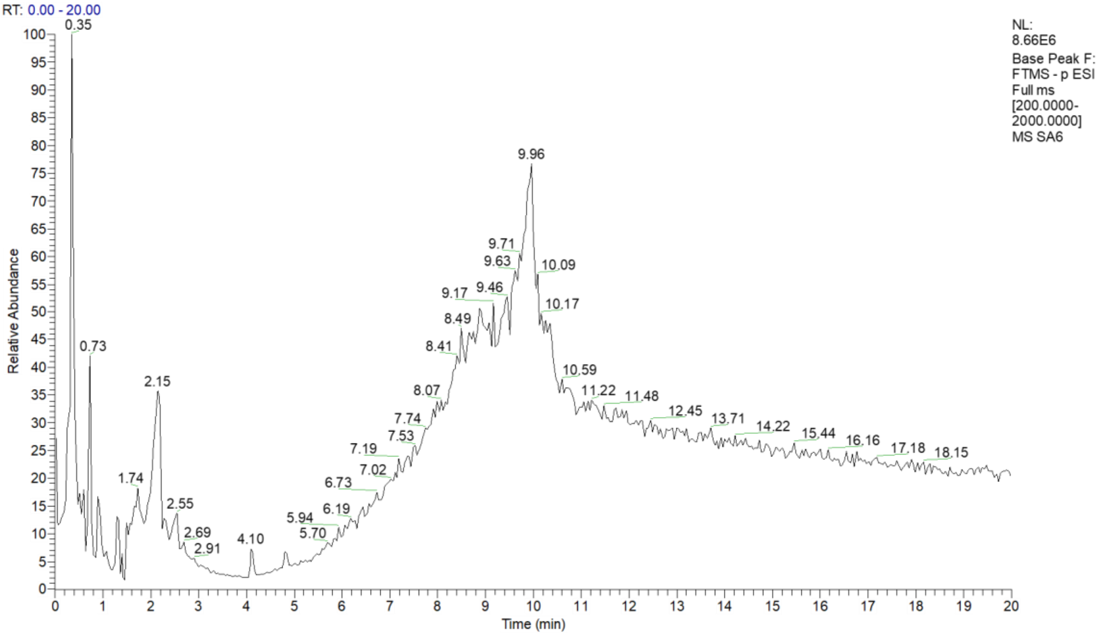
RT: 0.00 - 20.00
NL: 8.66E6
Base Peak F: FTMS - p ESI Full ms [200.0000-2000.0000] MS SA6
Relative Abundance
Time (min)
0.35
9.96
9.71
9.63
10.09
9.17
9.46
10.17
8.49
8.41
0.73
10.59
2.15
11.22
11.48
8.07
12.45
7.74
13.71
14.22
7.53
15.44
16.16
7.19
17.18
18.15
1.74
7.02
6.73
2.55
6.19
5.94
5.70
2.69
2.91
4.10

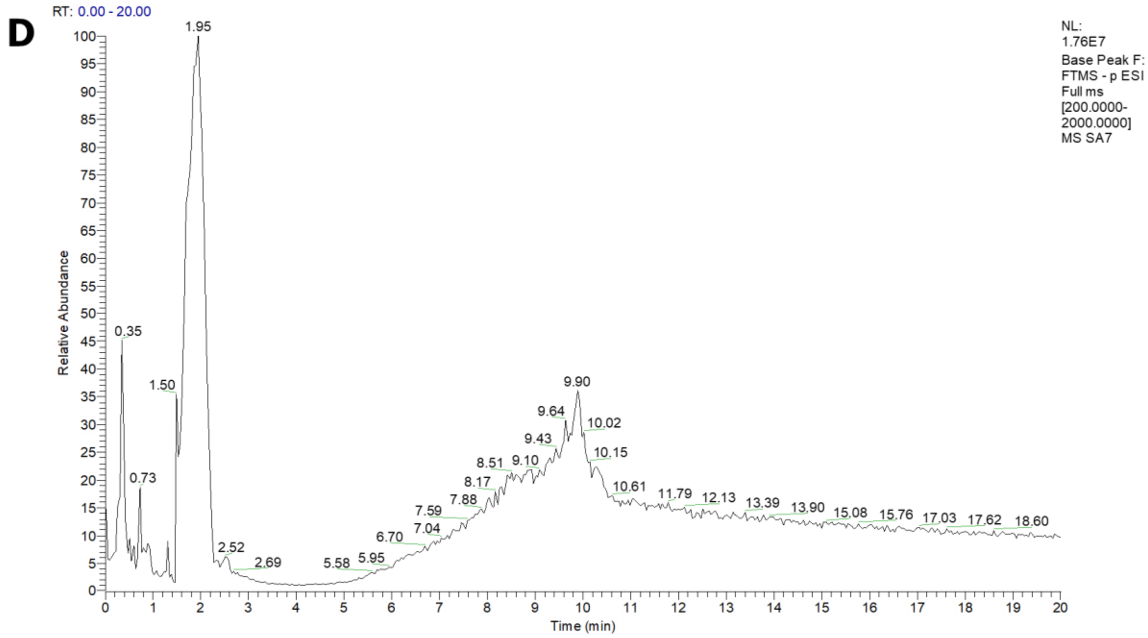

Figure S3. UHPLC–Q/Orbitrap/MS HRMS chromatograms with relative abundance and retention time (min) of (**A**) root; (**B**) stem bark; (**C**) dichloromethane leaf; and (**D**) methanol leaf extracts of *Lippia multiflora* obtained in negative mode electrospray ionization.

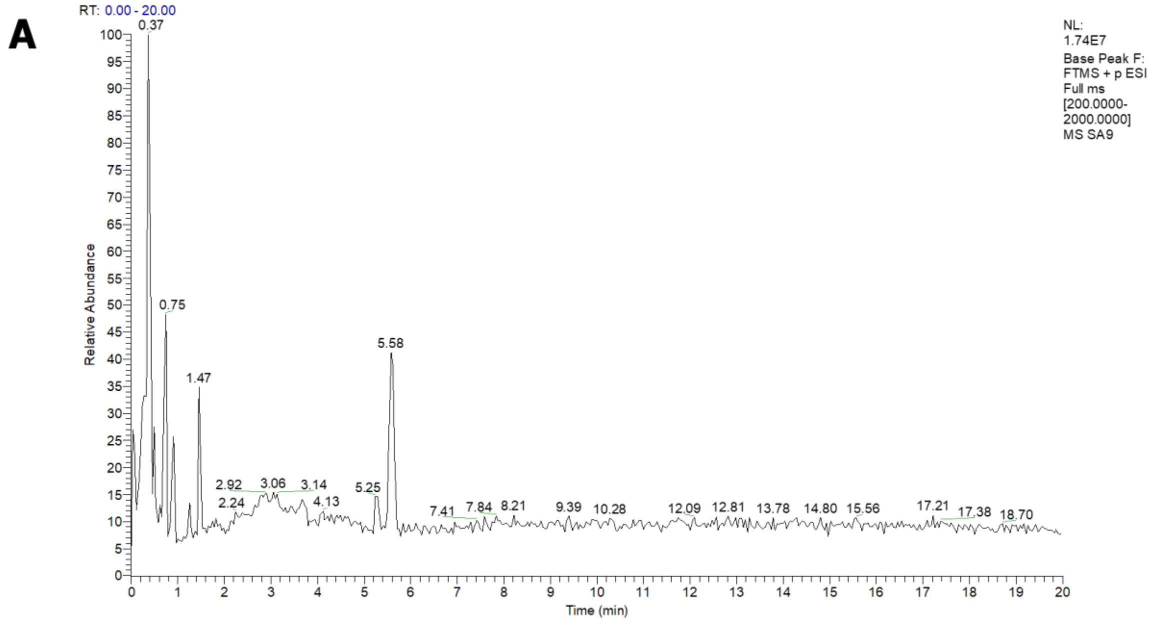

B

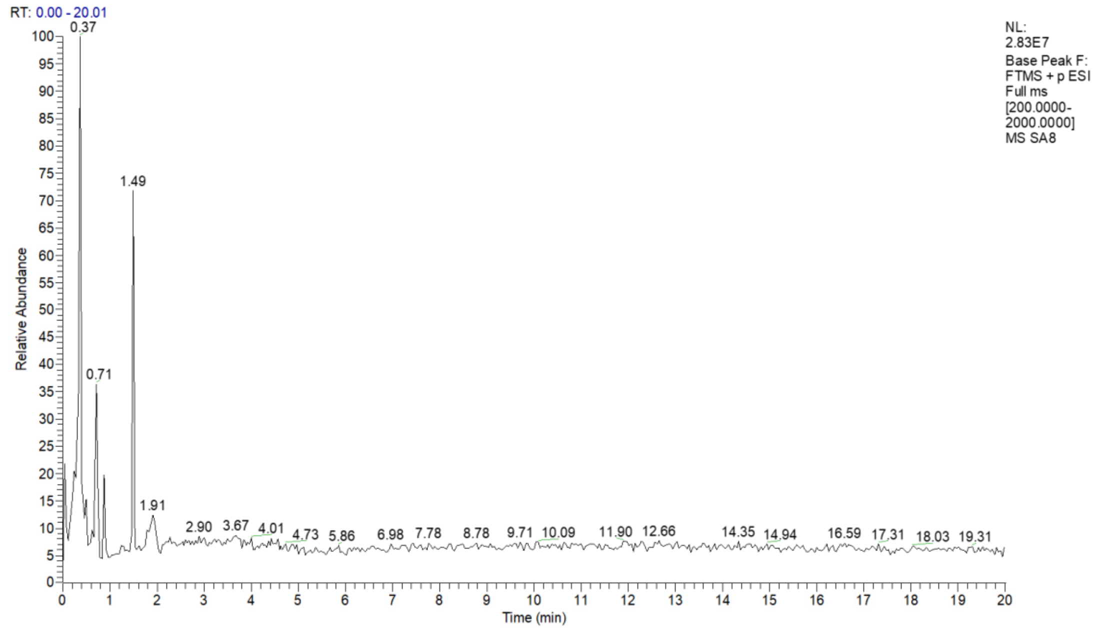
RT: 0.00 - 20.01
NL:
2.83E7
Base Peak F:
FTMS + p ESI
Full ms
[200.0000-
2000.0000]
MS SA8
0.37
1.49
0.71
1.91
2.90
3.67
4.01
4.73
5.86
6.98
7.78
8.78
9.71
10.09
11.90
12.66
14.35
14.94
16.59
17.31
18.03
19.31
Relative Abundance
Time (min)

C

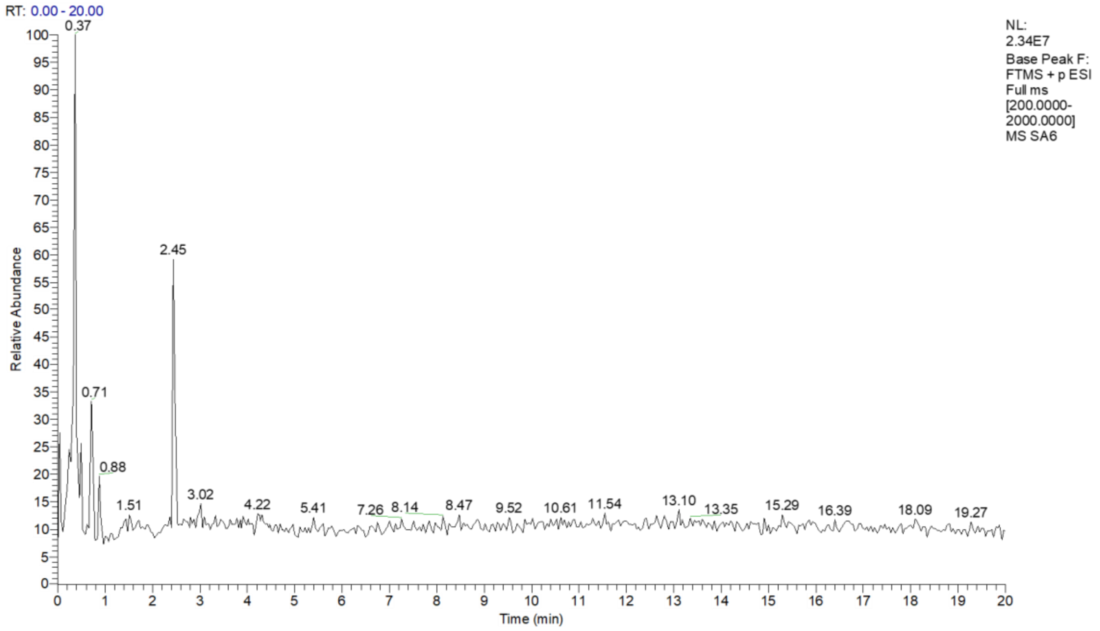
RT: 0.00 - 20.00
NL:
2.34E7
Base Peak F:
FTMS + p ESI
Full ms
[200.0000-
2000.0000]
MS SA6
0.37
2.45
0.71
0.88
1.51
3.02
4.22
5.41
7.26
8.14
8.47
9.52
10.61
11.54
13.10
13.35
15.29
16.39
18.09
19.27
Relative Abundance
Time (min)

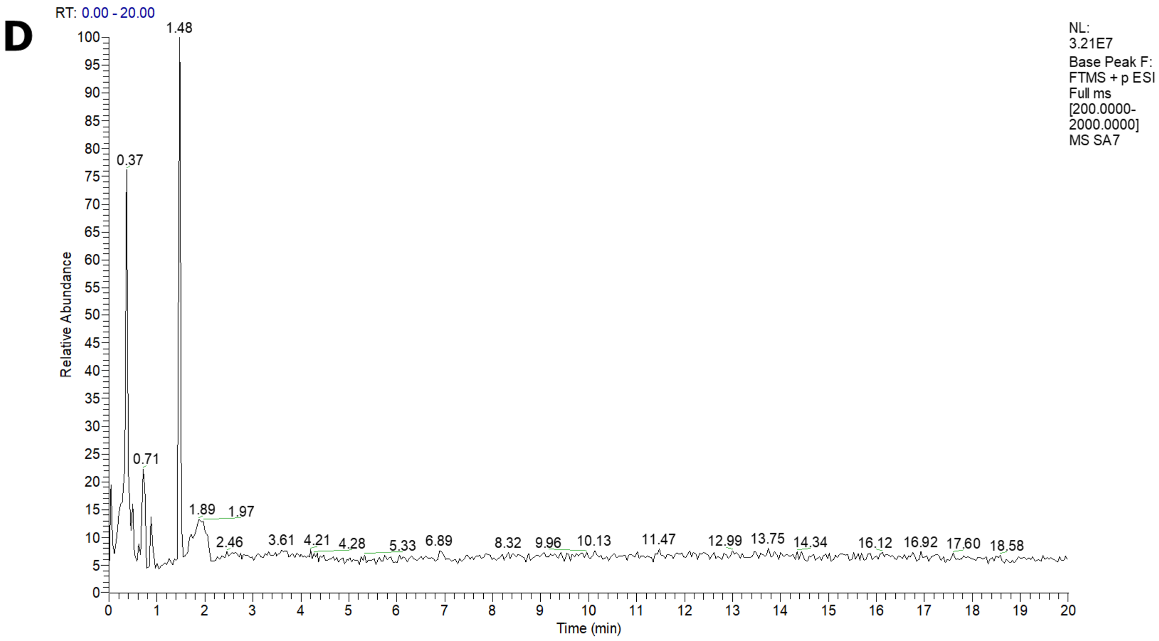

Figure S4. UHPLC–Q/Orbitrap/MS HRMS chromatograms with relative abundance and retention time (min) of (**A**) root; (**B**) stem bark; (**C**) dichloromethane leaf; and (**D**) methanol leaf extracts of *Lippia multiflora* obtained in positive mode electrospray ionization.

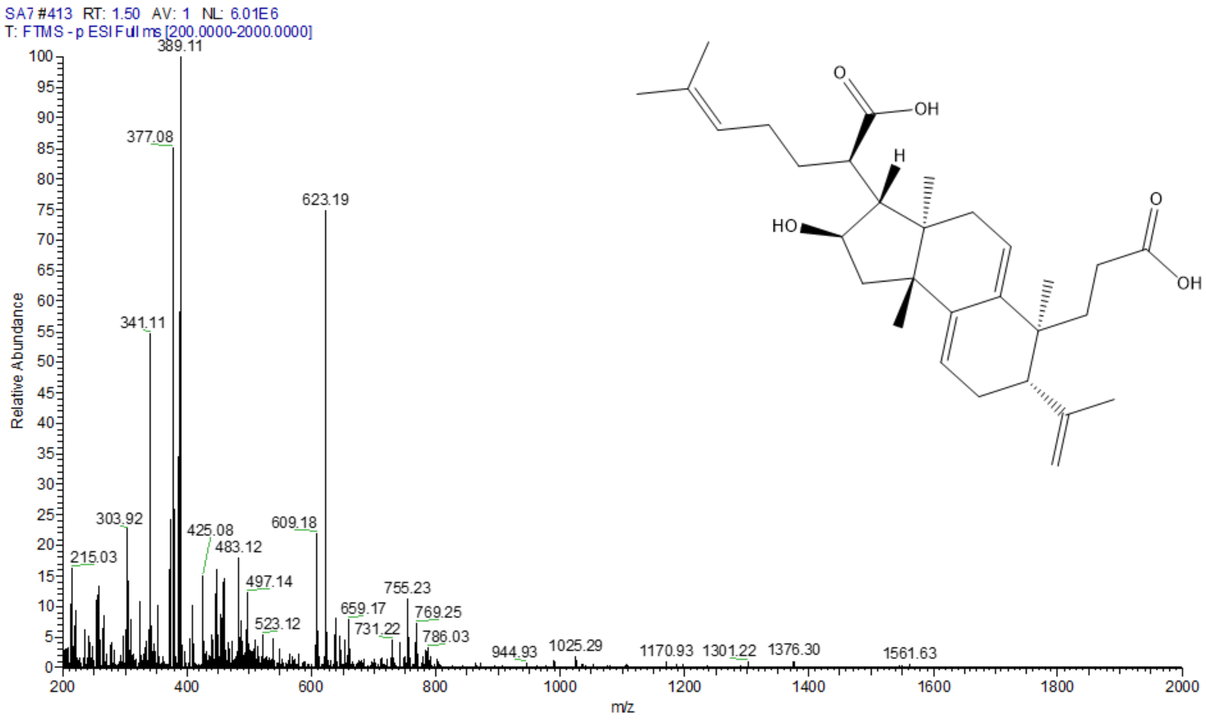

Figure S5. UHPLC–Q/Orbitrap/MS HRMS spectrum of poricoic acid B (**1**).

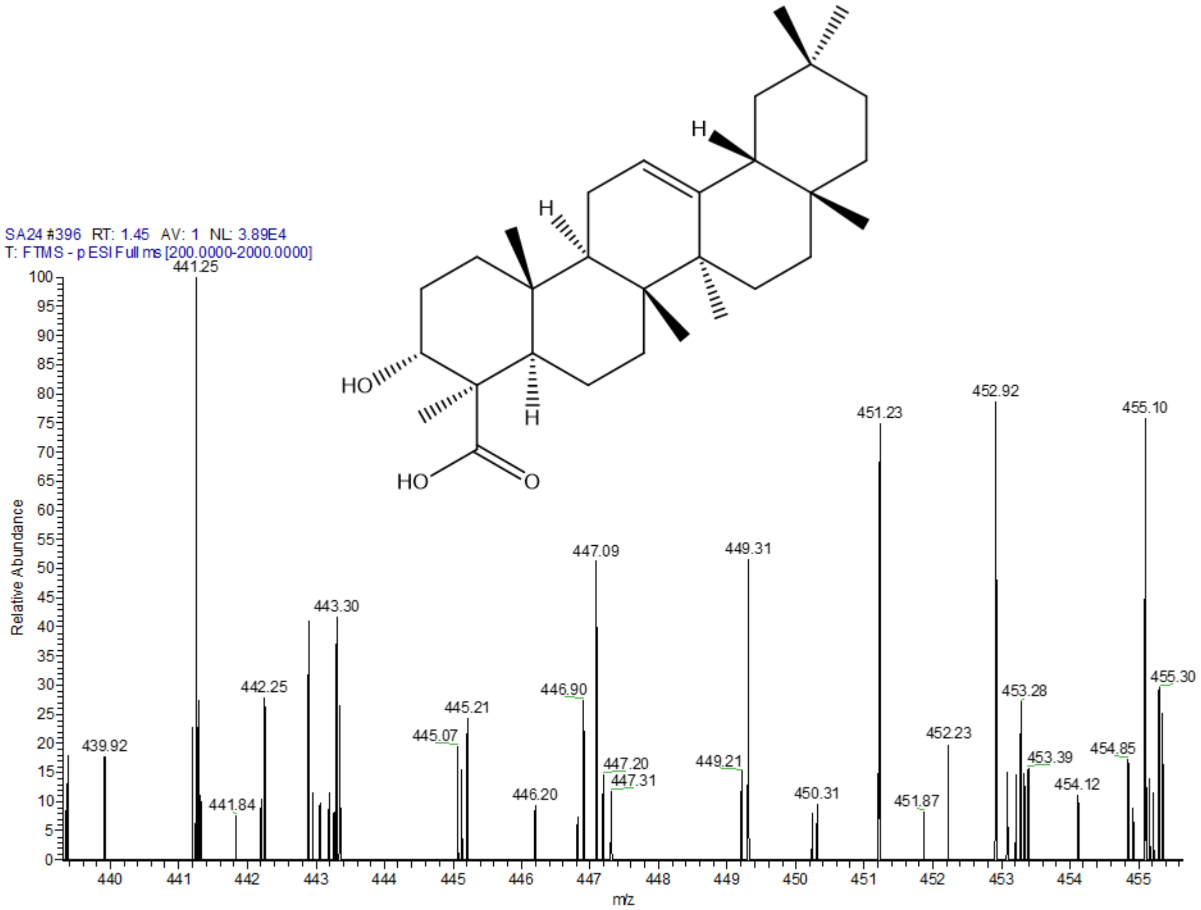

Figure S6. UHPLC–Q/Orbitrap/MS HRMS spectrum of boswellic acid alpha (**2**).

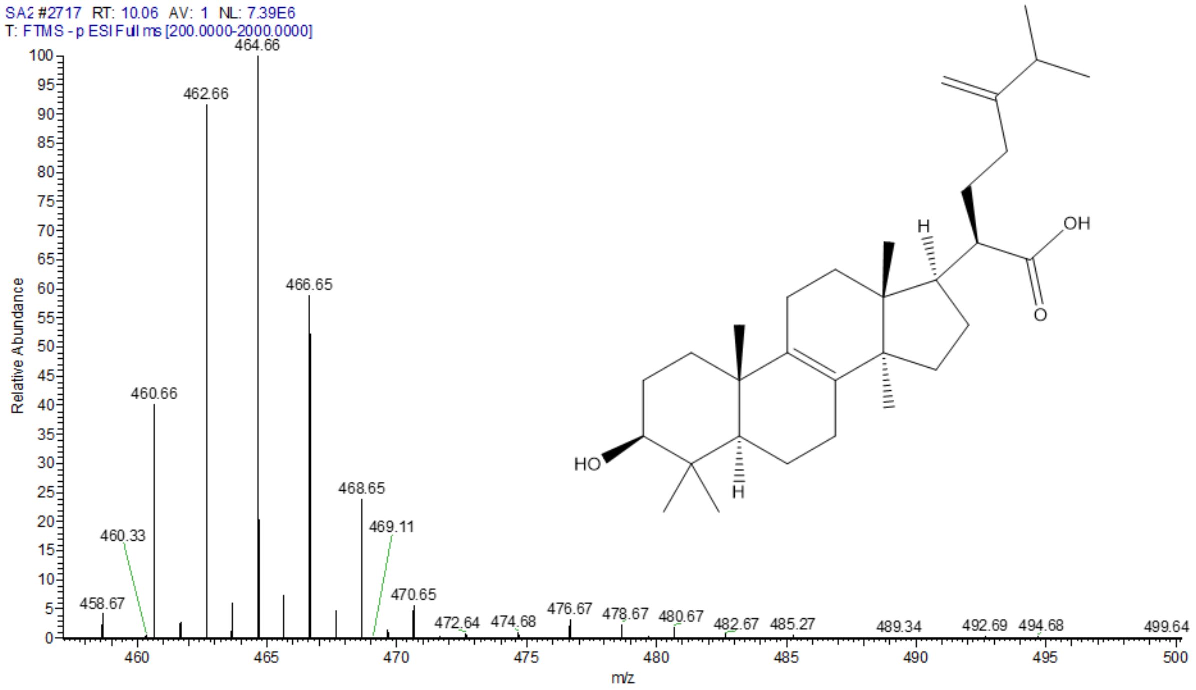

Figure S7. UHPLC–Q/Orbitrap/MS HRMS spectrum of eburicoic acid (**3**).

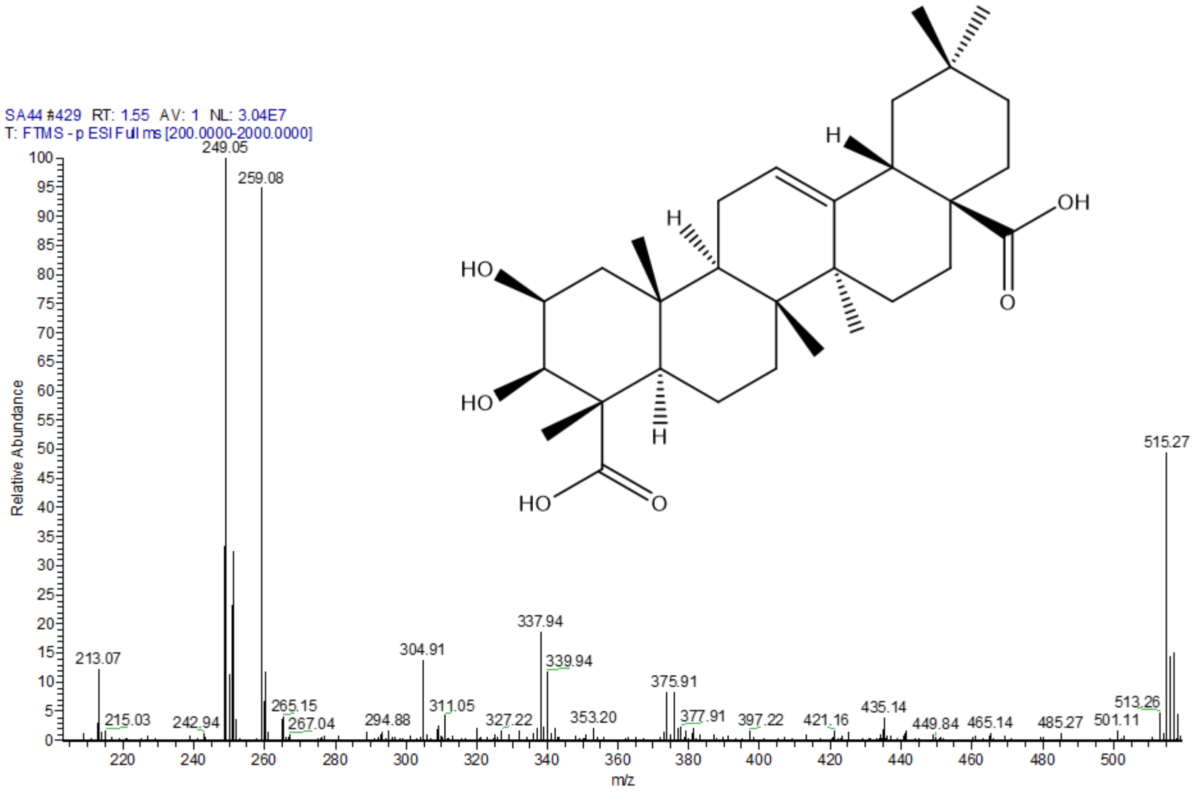

Figure S8. UHPLC–Q/Orbitrap/MS HRMS spectrum of medicagenic acid (**4**).

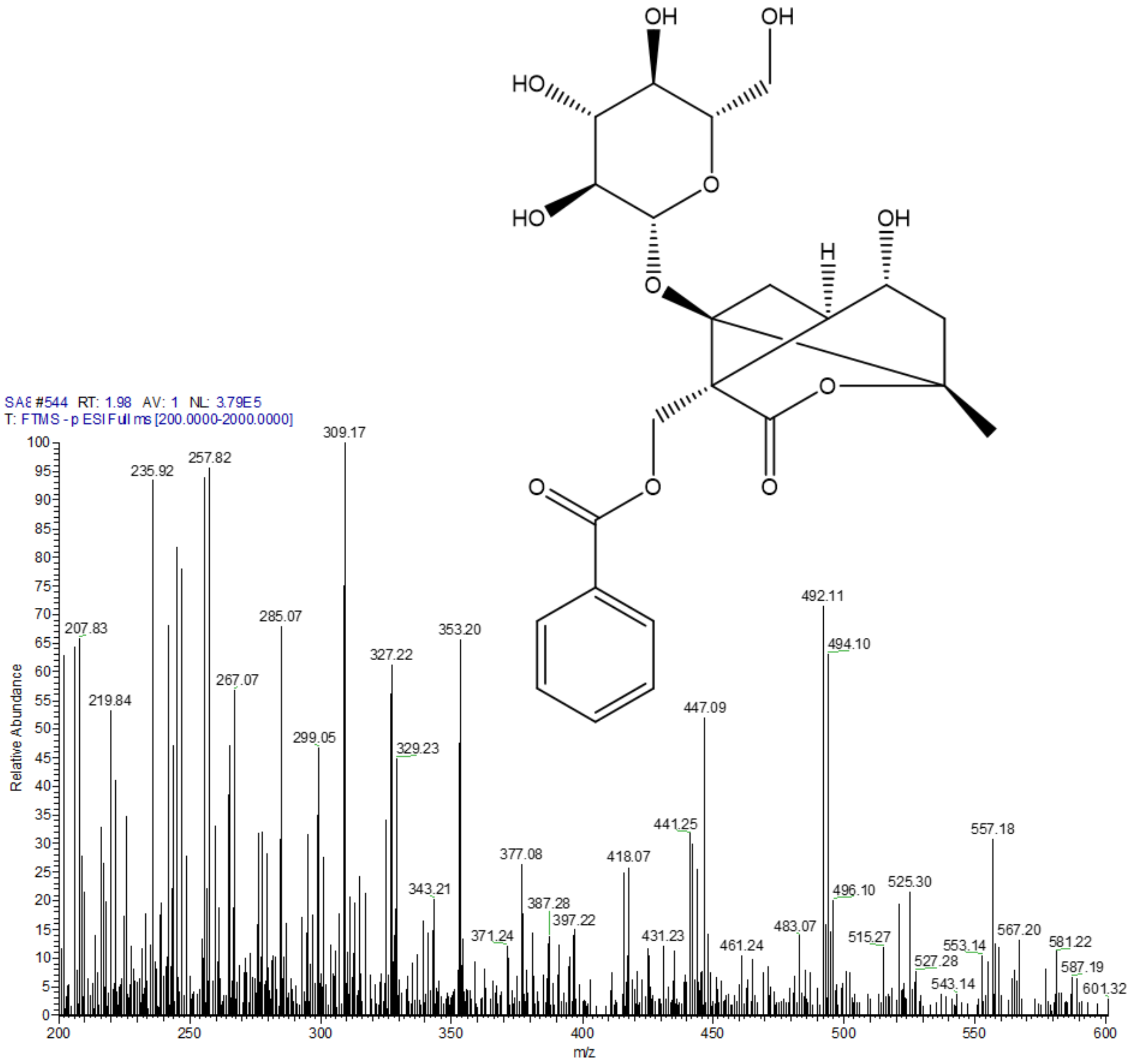

Figure S9. UHPLC–Q/Orbitrap/MS HRMS spectrum of albiflorin (**5**).

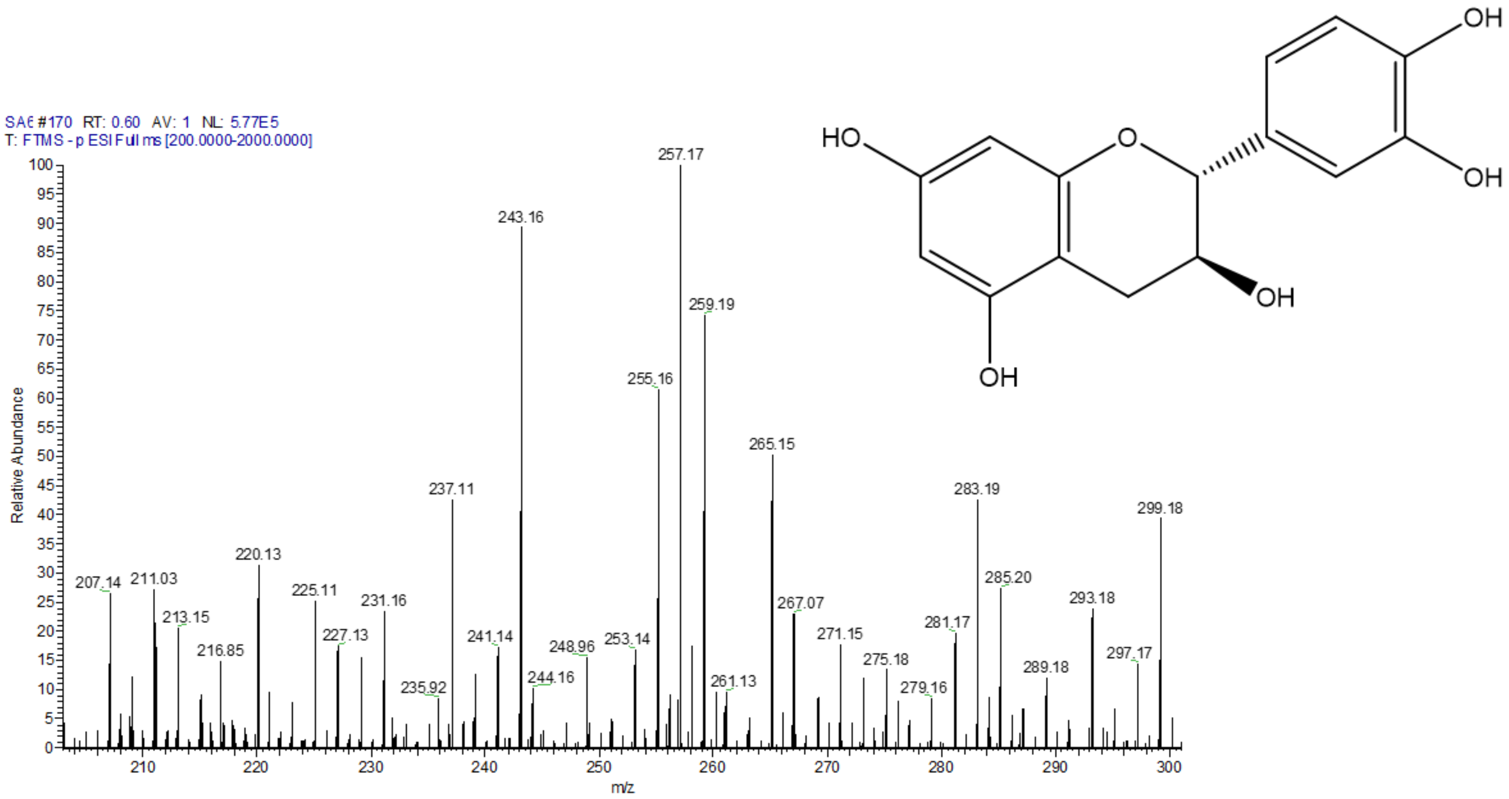

Figure S10. UHPLC–Q/Orbitrap/MS HRMS spectrum of catechin (**6**).

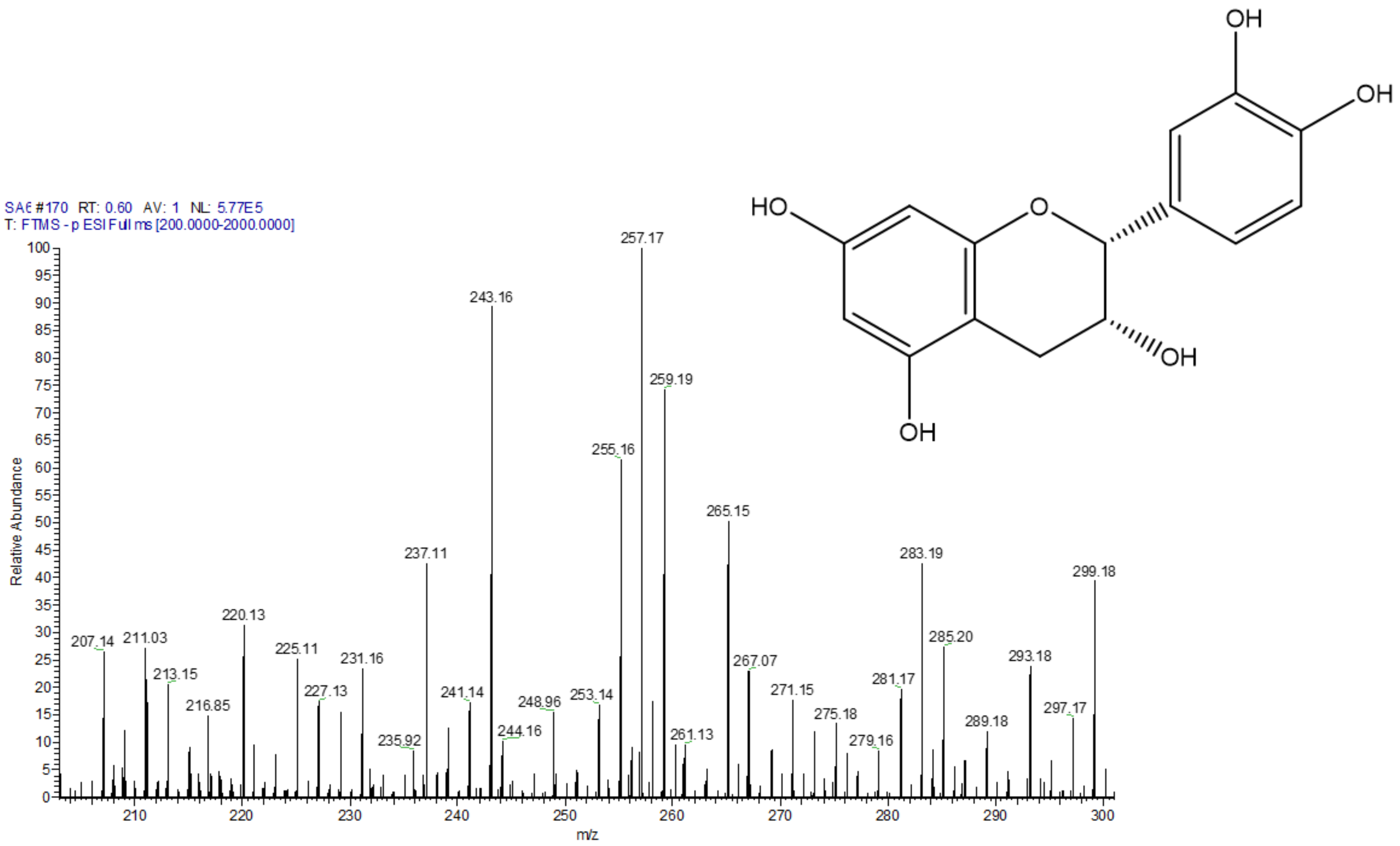

Figure S11. UHPLC–Q/Orbitrap/MS HRMS spectrum of epicatechin (**7**).

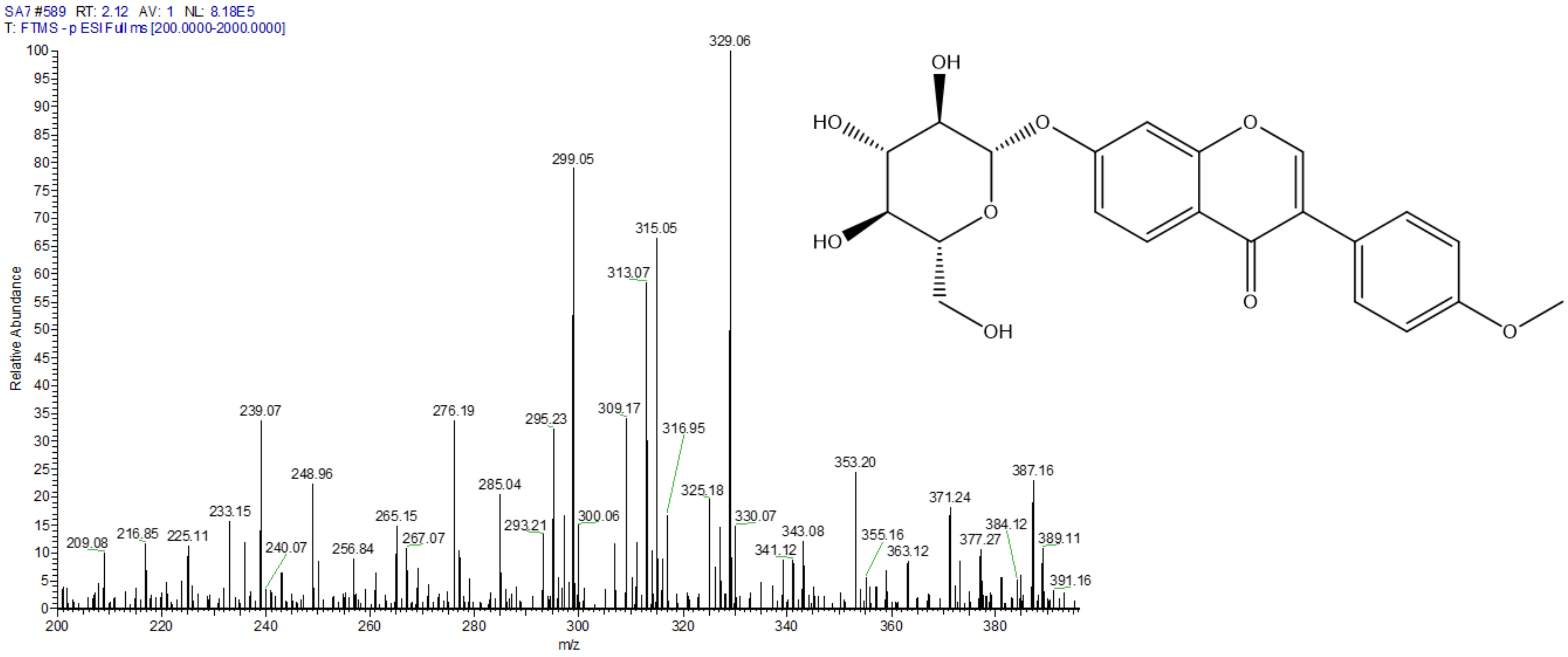

Figure S12. UHPLC–Q/Orbitrap/MS HRMS spectrum of formononetin-7-*O*-glucoside (Ononin) (**8**).

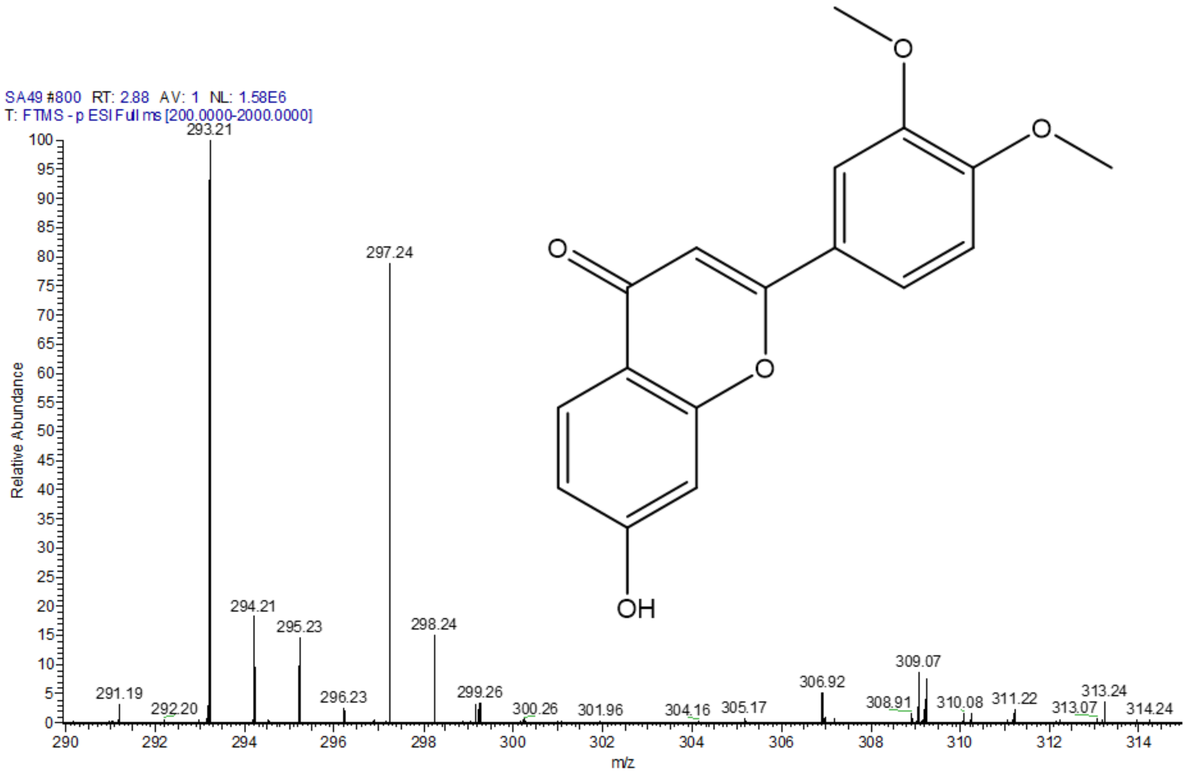

Figure S13. UHPLC–Q/Orbitrap/MS HRMS spectrum of 3',4'-dimethoxy-7-hydroxyflavone (**9**).

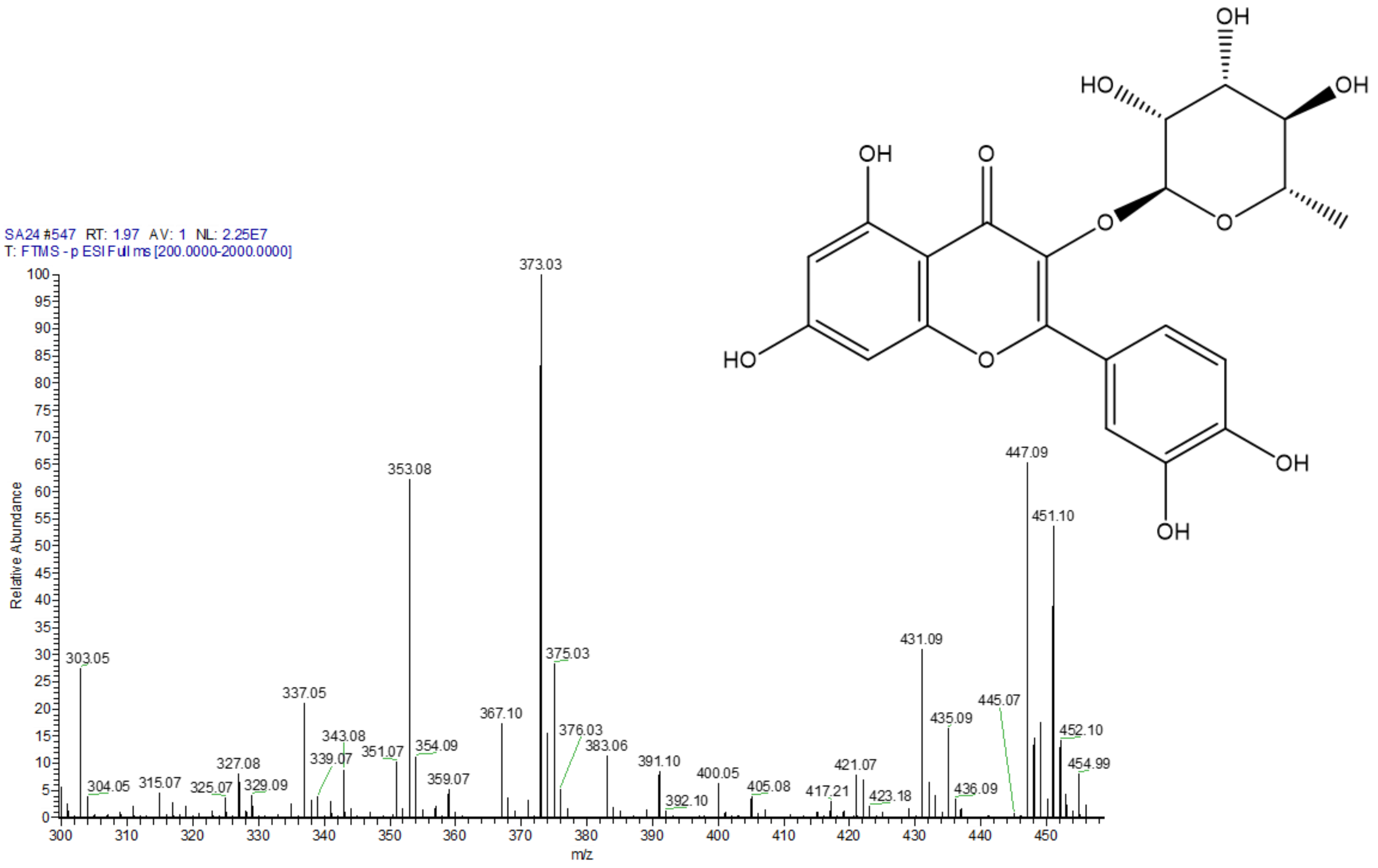

Figure S14. UHPLC–Q/Orbitrap/MS HRMS spectrum of quercitrin (**10**).

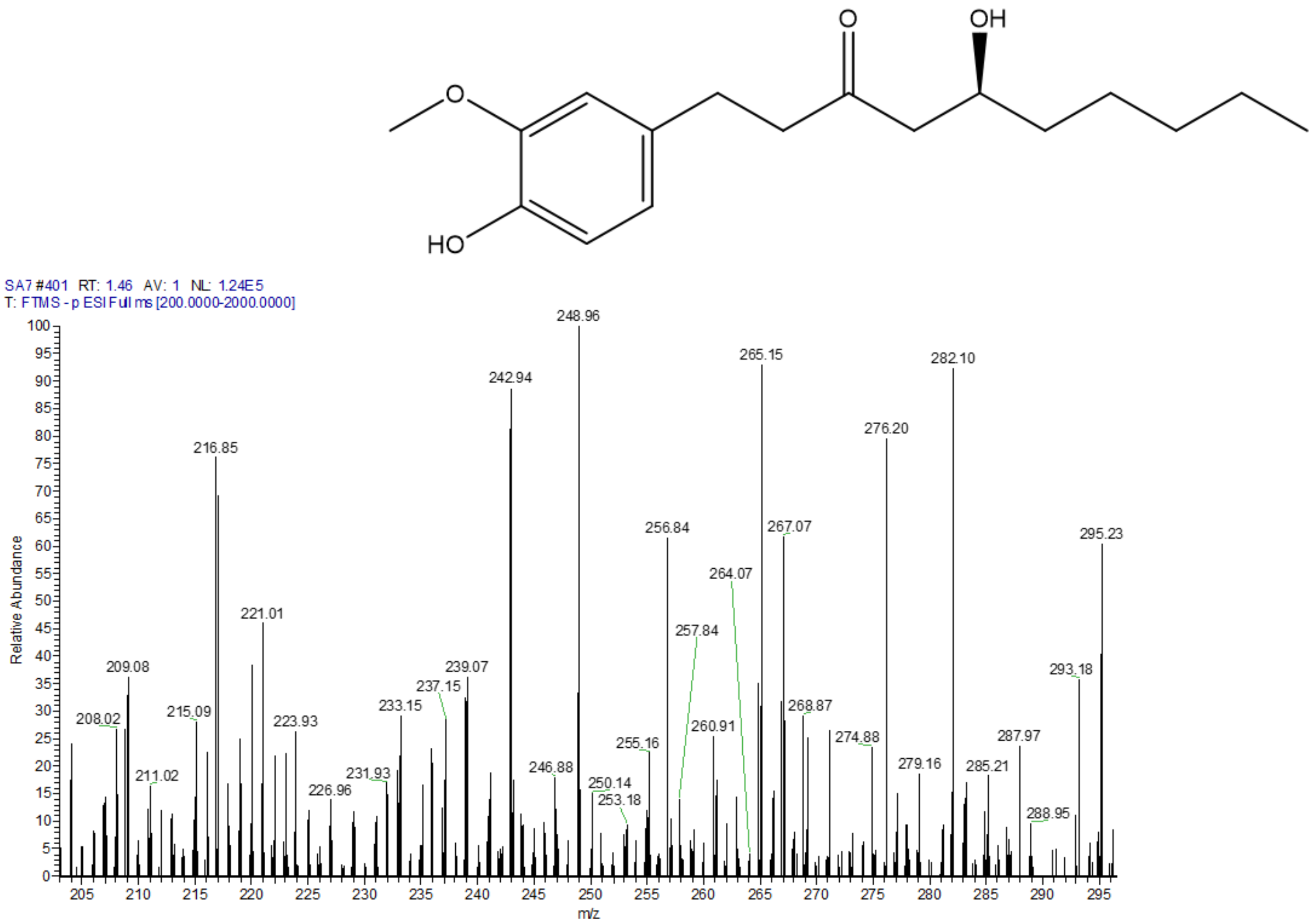

Figure S15. UHPLC–Q/Orbitrap/MS HRMS spectrum of 6-gingerol (**11**).

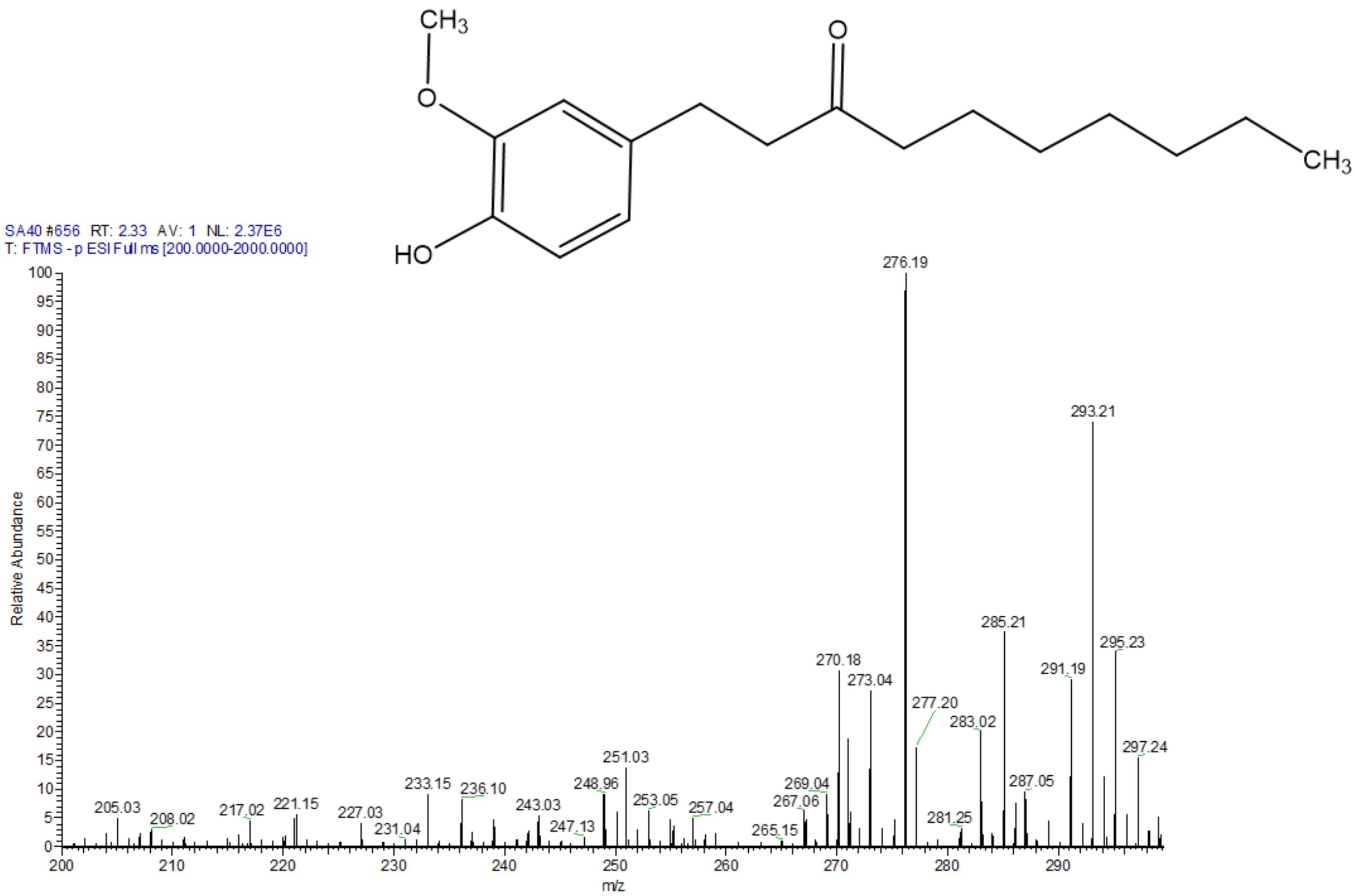

Figure S16. UHPLC–Q/Orbitrap/MS HRMS spectrum of 6-paradol (**12**).

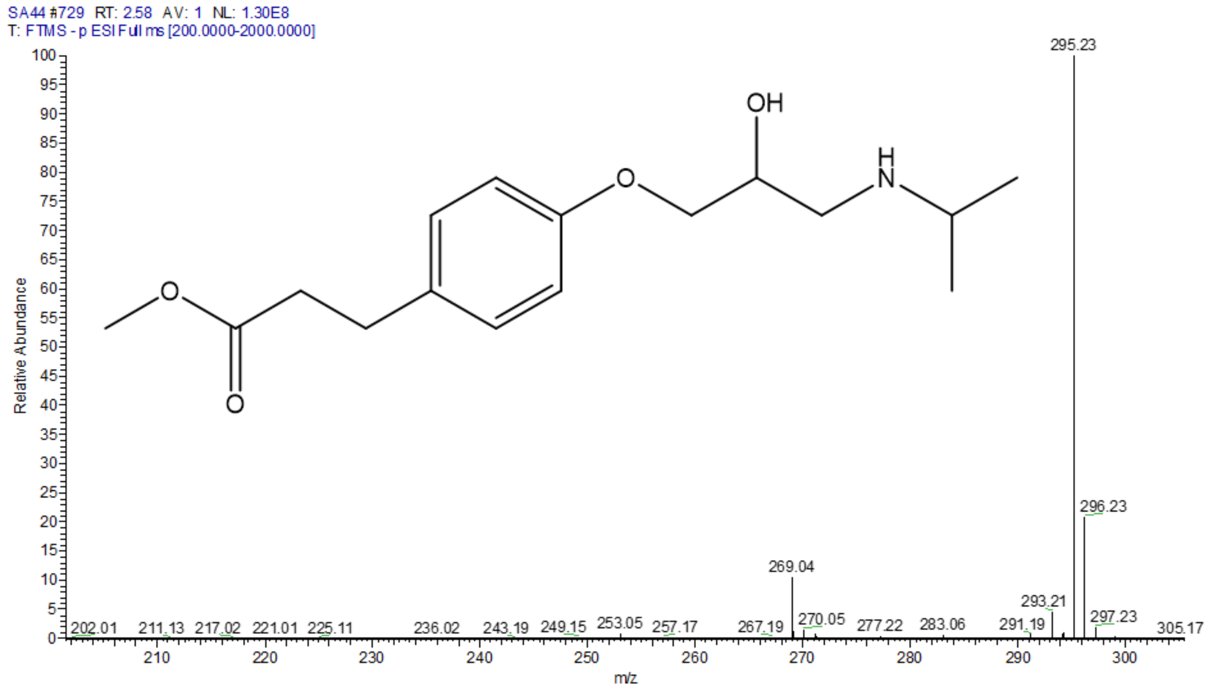

Figure S17. UHPLC–Q/Orbitrap/MS HRMS spectrum of esmolol (**13**).

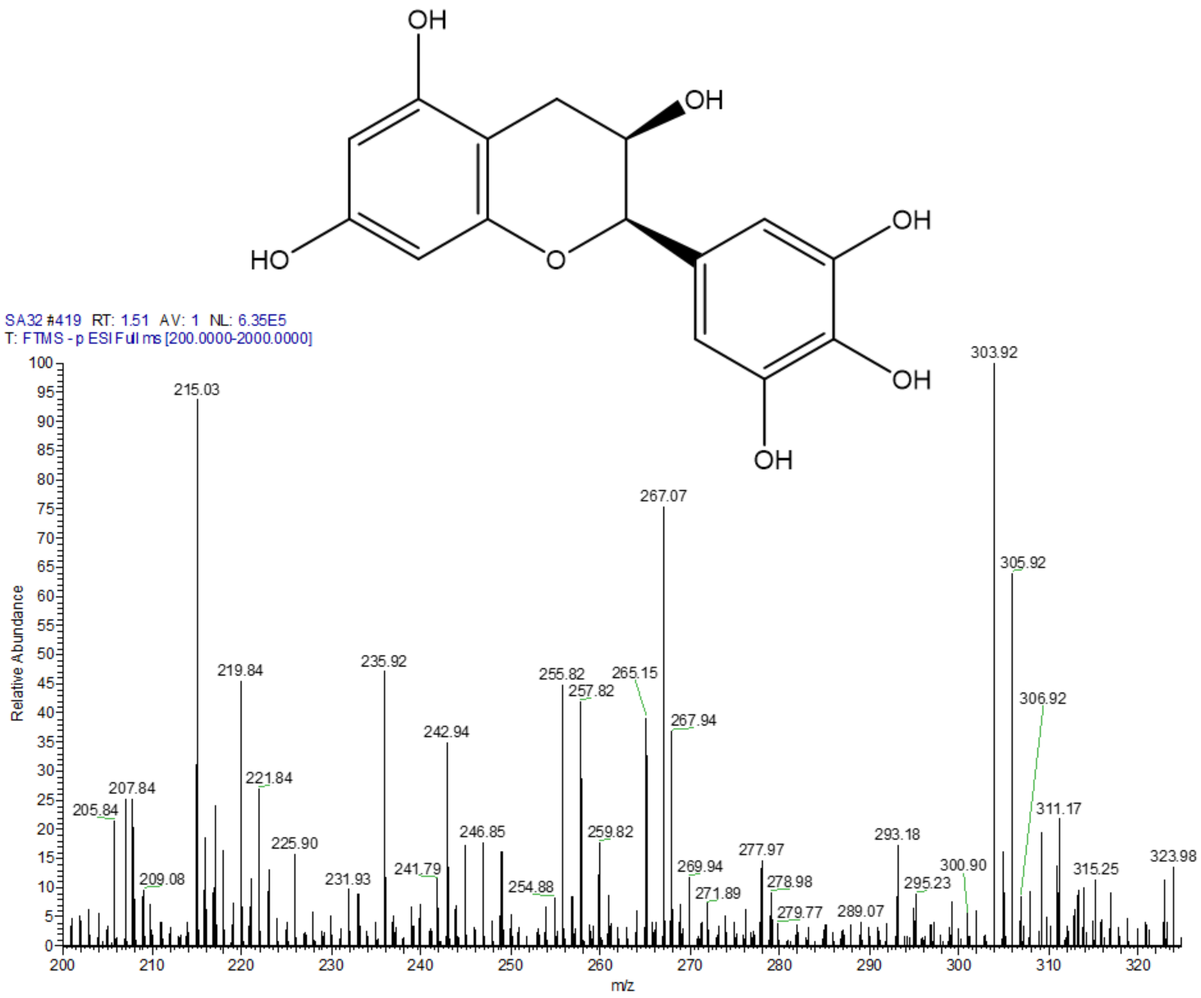

Figure S18. UHPLC–Q/Orbitrap/MS HRMS spectrum of (-)-epigallocatechin (**14**).

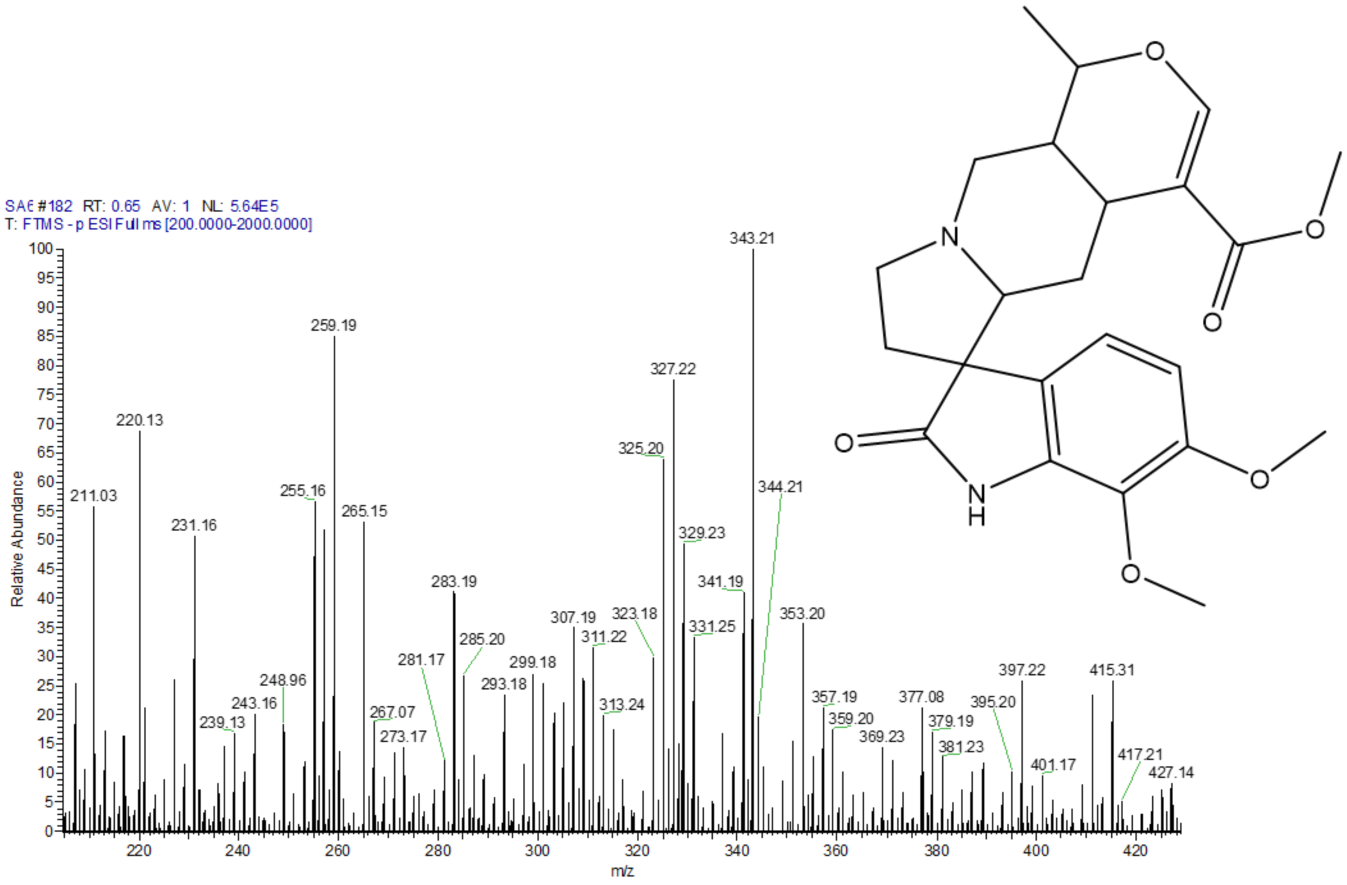

Figure S19. UHPLC–Q/Orbitrap/MS HRMS spectrum of isomajdine (**15**).

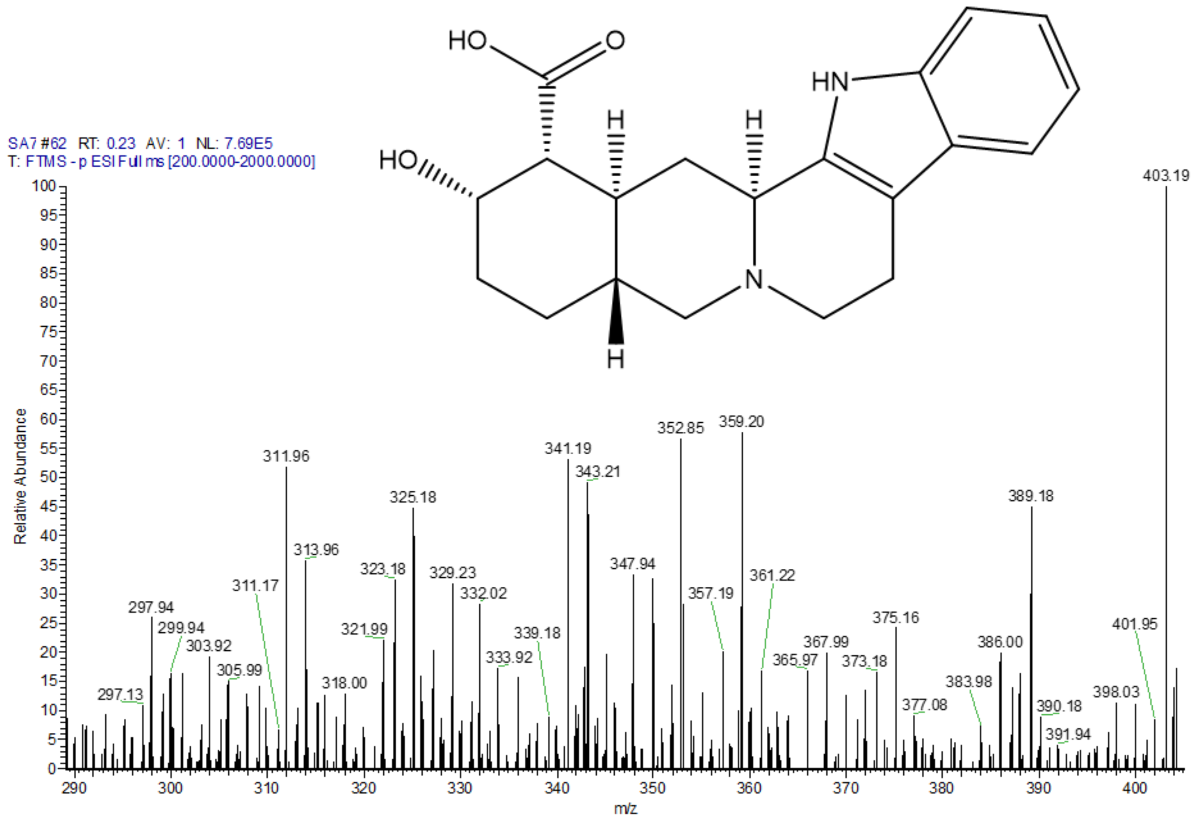

Figure S20. UHPLC–Q/Orbitrap/MS HRMS spectrum of yohimbinic acid (**16**).

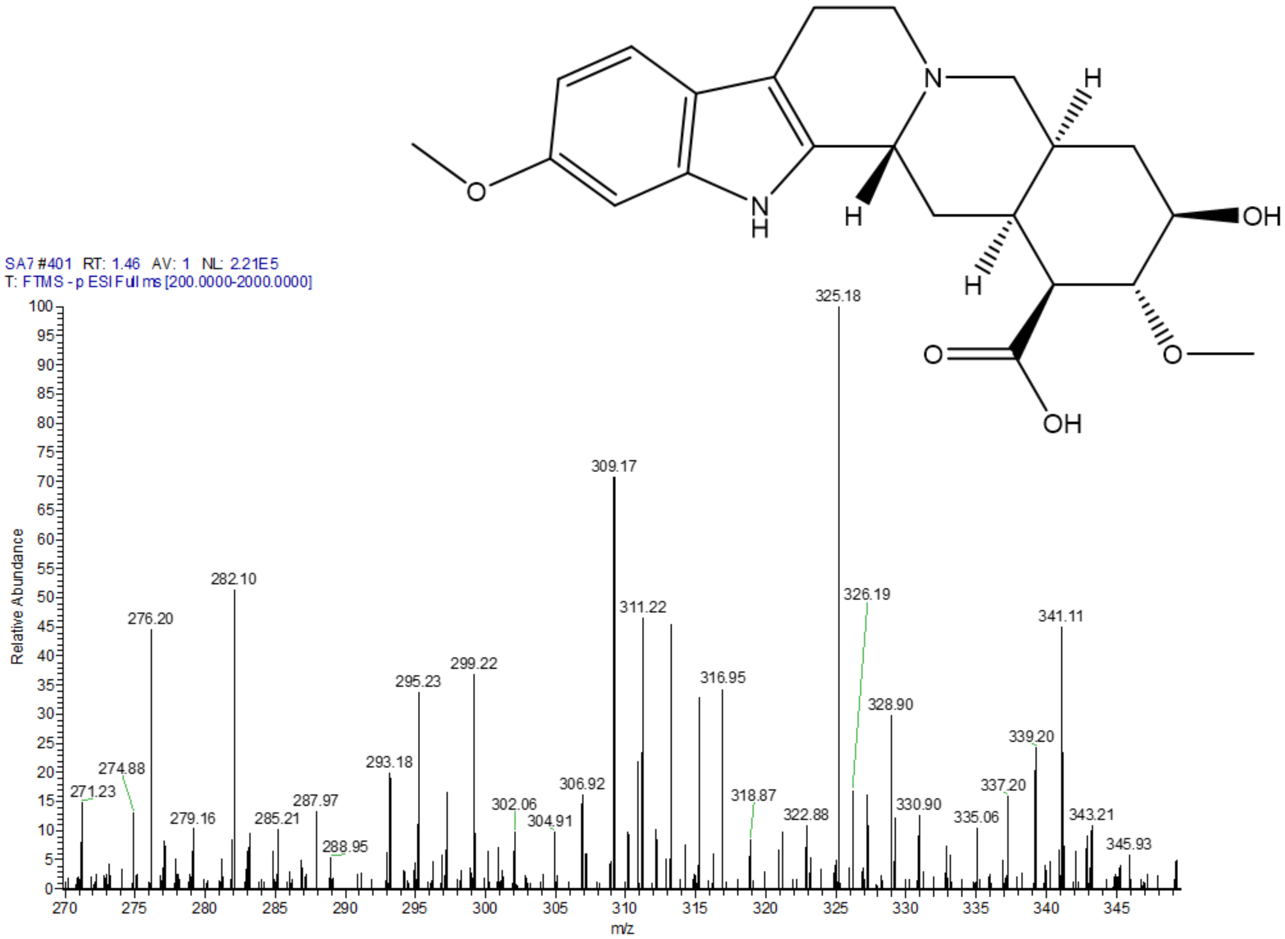

Figure S21. UHPLC–Q/Orbitrap/MS HRMS spectrum of reserpic acid (**17**).

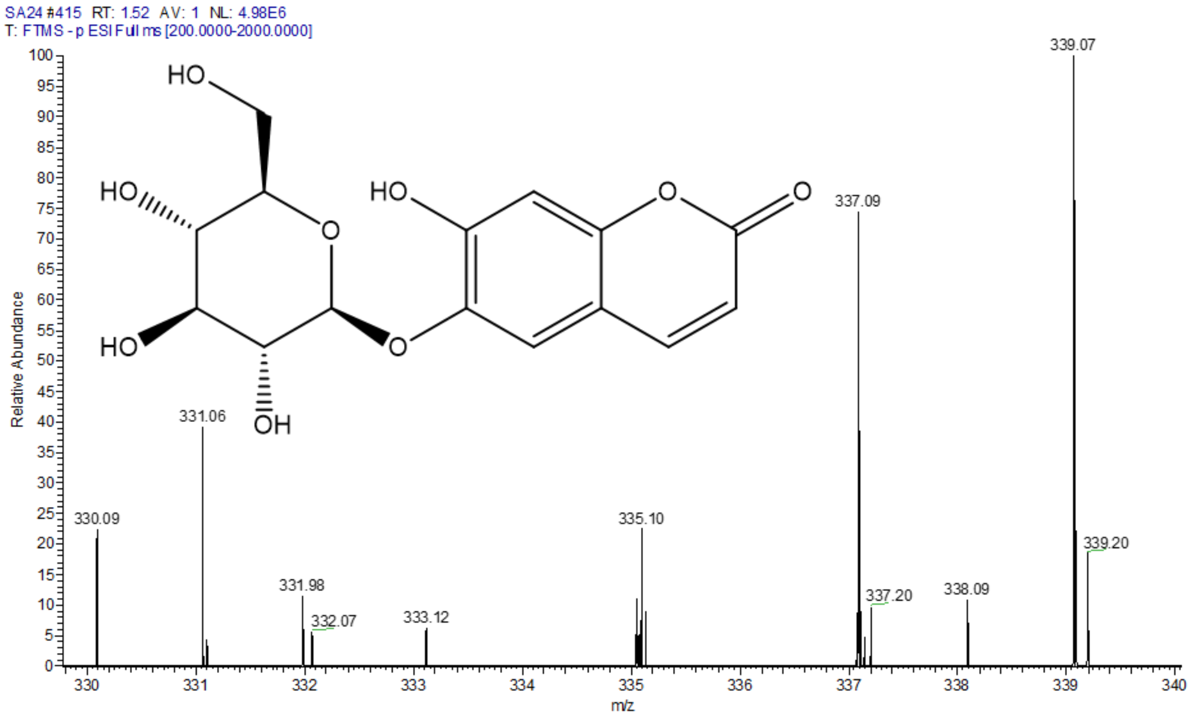

Figure S22. UHPLC–Q/Orbitrap/MS HRMS spectrum of 6,7-dihydroxycoumarin-6-glucoside (Esculin) (**18**).

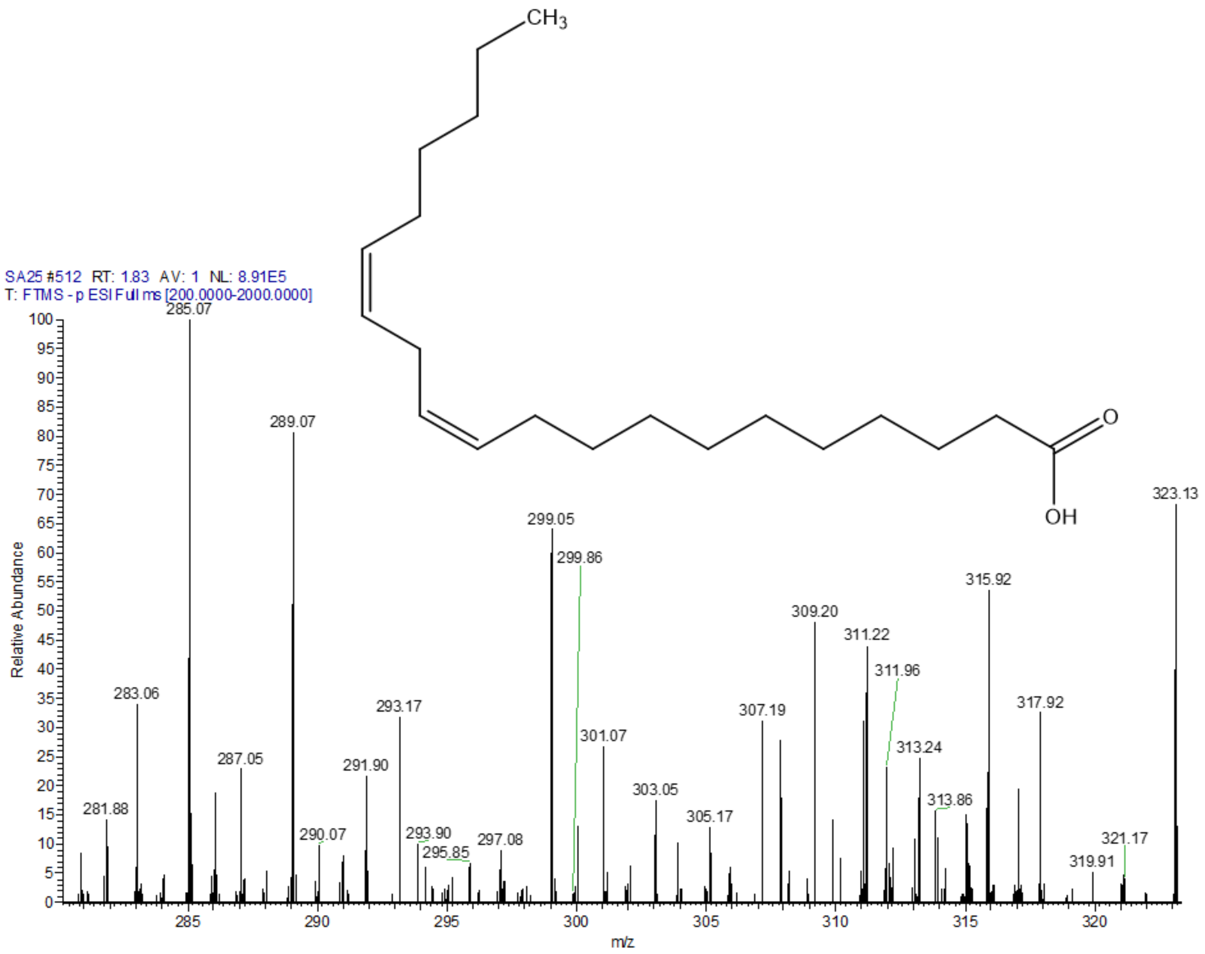

Figure S23. UHPLC–Q/Orbitrap/MS HRMS spectrum of eicosadieneoic acid (**19**).

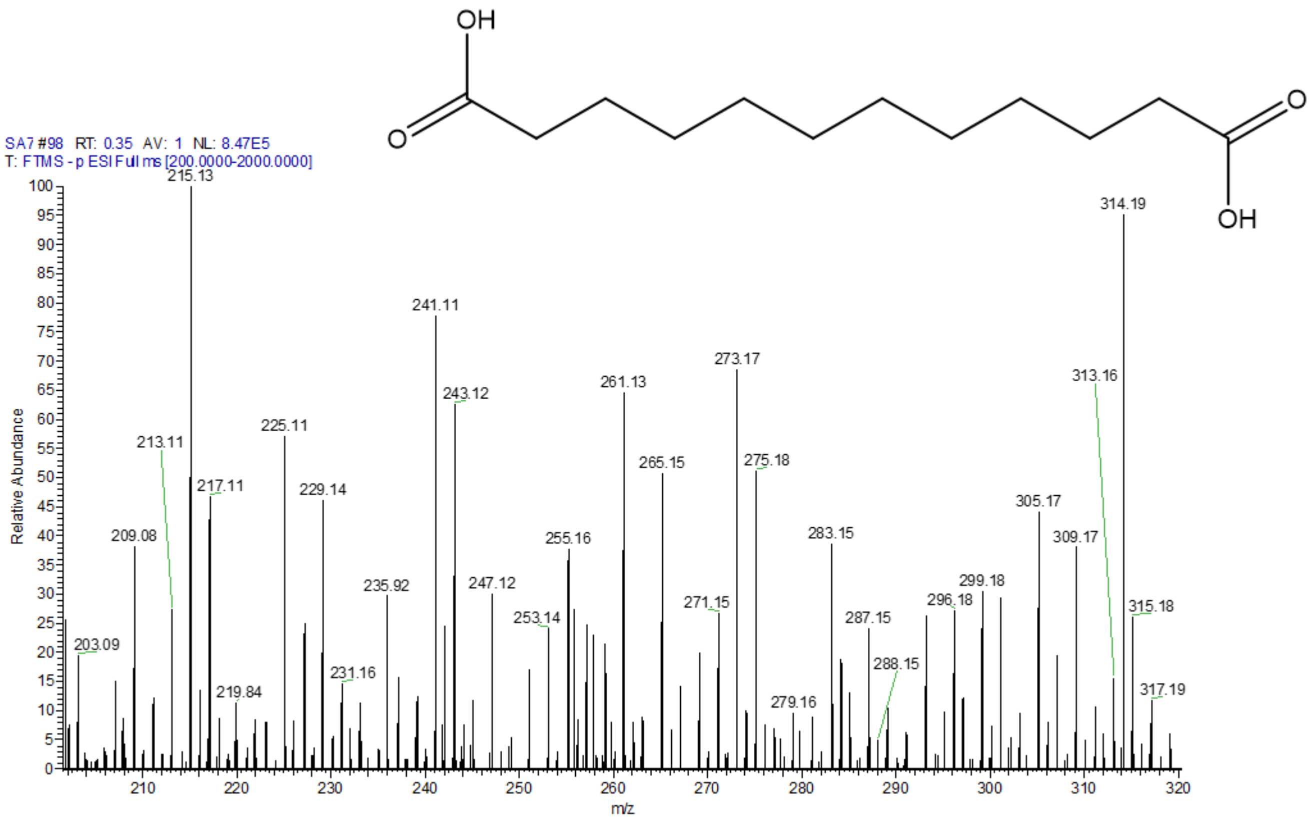

Figure S24. UHPLC–Q/Orbitrap/MS HRMS spectrum of dodecanedioic acid (**20**).
